# Bayesian optimization with evolutionary and structure-based regularization for directed protein evolution

**DOI:** 10.1186/s13015-021-00195-4

**Published:** 2021-07-01

**Authors:** Trevor S. Frisby, Christopher James Langmead

**Affiliations:** Computational Biology Department, School of Computer Science, Carnegie Mellon University, 5000 Forbes Ave, Pittsburgh, PA 15213 USA

**Keywords:** Protein design, Bayesian optimization, Regularization, Directed evolution, Rational design, Gaussian process regression, Protein language model, Active learning

## Abstract

**Background:**

Directed evolution (DE) is a technique for protein engineering that involves iterative rounds of mutagenesis and screening to search for sequences that optimize a given property, such as binding affinity to a specified target. Unfortunately, the underlying optimization problem is under-determined, and so mutations introduced to improve the specified property may come at the expense of unmeasured, but nevertheless important properties (ex. solubility, thermostability, etc). We address this issue by formulating DE as a regularized Bayesian optimization problem where the regularization term reflects evolutionary or structure-based constraints.

**Results:**

We applied our approach to DE to three representative proteins, GB1, BRCA1, and SARS-CoV-2 Spike, and evaluated both evolutionary and structure-based regularization terms. The results of these experiments demonstrate that: (i) structure-based regularization usually leads to better designs (and never hurts), compared to the unregularized setting; (ii) evolutionary-based regularization tends to be least effective; and (iii) regularization leads to better designs because it effectively focuses the search in certain areas of sequence space, making better use of the experimental budget. Additionally, like previous work in Machine learning assisted DE, we find that our approach significantly reduces the experimental burden of DE, relative to model-free methods.

**Conclusion:**

Introducing regularization into a Bayesian ML-assisted DE framework alters the exploratory patterns of the underlying optimization routine, and can shift variant selections towards those with a range of targeted and desirable properties. In particular, we find that structure-based regularization often improves variant selection compared to unregularized approaches, and never hurts.

**Supplementary Information:**

The online version contains supplementary material available at 10.1186/s13015-021-00195-4.

## Introduction

The field of protein engineering seeks to design molecules with novel or improved properties [1]. The primary techniques used in protein engineering fall into two broad categories: *rational design* [2] and *directed evolution* (DE) [3]. Rational design uses model-driven in silico combinatorial searches to identify promising candidate designs, which are then synthesized and tested experimentally. Directed evolution, in contrast, involves iterative rounds of saturation mutagenesis at select residue positions, followed by in vitro or in vivo screening for desirable traits. The most promising sequences are then isolated and used to seed the next round of mutagenesis.

Traditionally, directed evolution is a model-free approach. That is, computational models are not used to guide or simulate mutagenesis. Recently, however, a technique for incorporating Machine learning (ML) into the DE workflow was introduced [4]. Briefly, this ML-assisted form of DE uses the screening data from each round to update a model that predicts the effects of mutations on the property being optimized. The mutagenesis step in the next round of DE is then biased towards generating sequences with the desired property under the model, as opposed to generating a uniformly random sample. ML-assisted DE has been shown to reduce the number of rounds needed to find optimal sequences, relative to traditional (i.e., model-free) DE [4].

Significantly, the models learned in ML-assisted DE are *myopic* in the sense that they only consider the relationship between a limited set of residues (ex. those in a binding interface) and the screened trait (ex binding affinity). Thus, DE may improve the measured trait at the expense of those that are unmeasured, but nevertheless important (ex. thermostability, solubility, subcellular localization, etc). The primary goal of this paper is to introduce an enhanced version of ML-assisted DE that is biased towards native-like designs, while optimizing the desired trait. By ‘native’ we mean that the optimized design still has high probability under a generative model of protein sequences, or is predicted to be thermodynamically stable, according to a given energy function. The intuition behind this approach is that any sequence with these properties is likely to respect factors that are not directly accounted for by the fitness model, such as epistatic interactions between the mutated residues and the rest of the protein [5], among others.

Our method performs Bayesian optimization [6] and incorporates a regularization factor derived from either a generative model of protein sequence or an in silico prediction of structural thermodynamic stability. In this paper, we refer to these as “Evolutionary” or “Structure-based” regularization factors, respectively. Our method is agnostic with respect to the means by which the regularization factors are computed. For example, we evaluated three distinct generative models of protein sequences, including a contextual deep transformer language model [7], a Markov Random Field (MRF) generated by the gremlin algorithm [8], and profile Hidden Markov Model (HMMs) [9]. For structural thermodynamic stability, we use the FoldX protein design Suite [10] to calculate changes in Gibb’s free energy ($\Delta \Delta G$) associated with new designs.

We first demonstrate our method by re-designing the B1 domain of streptococcal protein G (GB1) at four residues to maximize binding affinity to the IgG Fc receptor. Next, using data obtained from deep-mutational scans, we use ML-assisted DE to investigate the factors governing the relationships between sequence and clinically relevant phenotypes. Specifically, we (i) identify variants of the RING domain of the BRCA1 protein for which the activity of tumor suppressor gene E3 ubiquitin ligase is maximal, and (ii) identify variants of the receptor binding domain of the SARS-CoV2 Spike protein that optimize binding affinity to the ACE2 receptor. Our results on these three targets demonstrate that a structure-based regularization term usually leads to better designs than the unregularized version, and never hurts. The results using an evolutionary-based regularization are mixed; it leads to better designs for GB1, but worse designs for BRCA1. We also demonstrate that a Bayesian approach to ML-assisted DE outperforms the (non-Bayesian) approach introduced in [4]. Specifically, we show that our approach reduces the wet-lab burden to identify optimal GB1 designs by $67\%$, relative to the results presented in [4] on the same data.

## Background

### Directed protein evolution

**Fig. 1:**
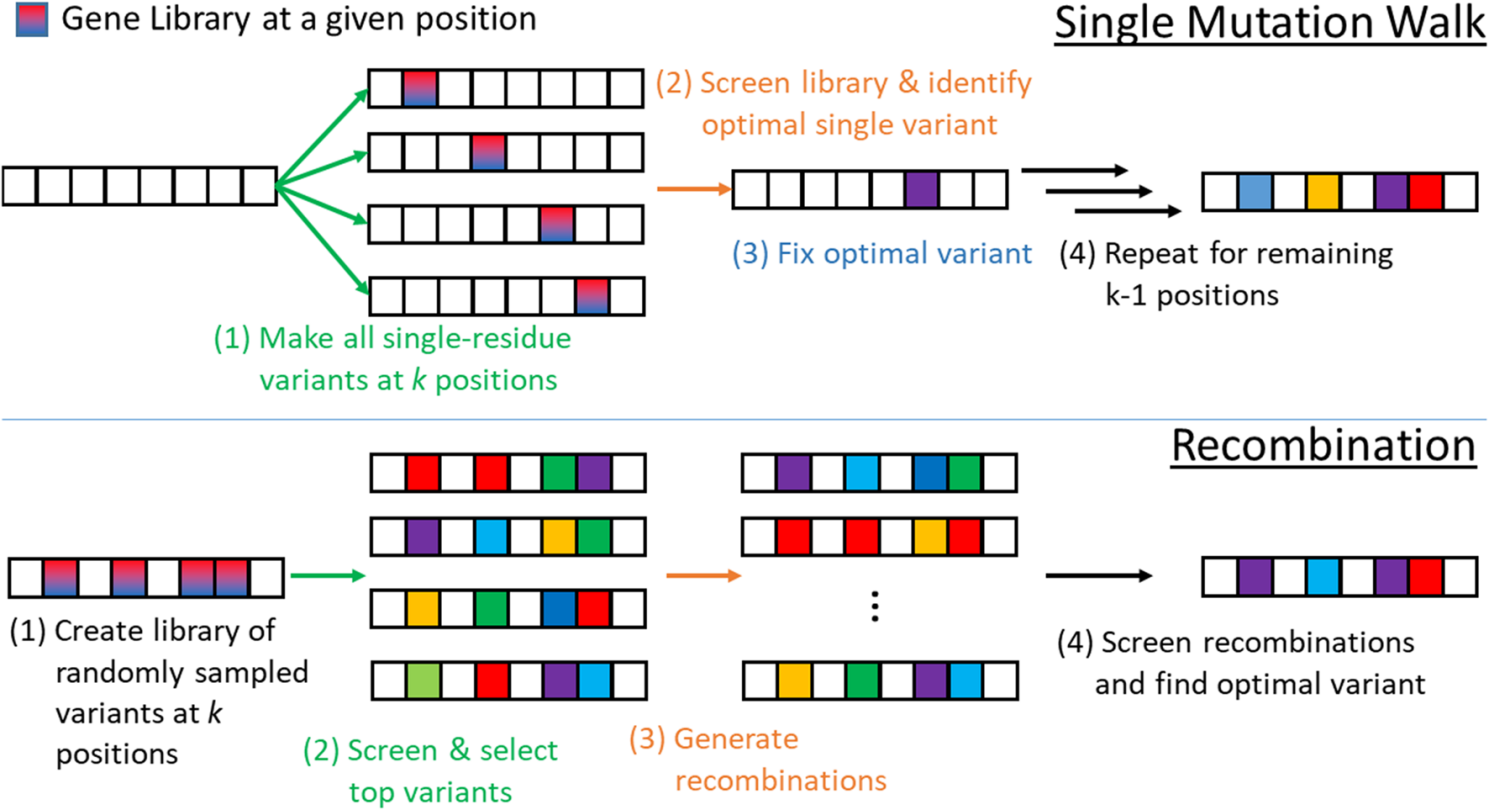
Traditional, model-free approaches to directed evolution: (*Top*) The ‘single mutation walk’ approach to directed evolution. The library of variants is the union of *k* libraries created by performing saturation mutagenesis at a single location. The resulting library, therefore, has 20*k* variants. The library is screened to find the single variant that optimizes the measured trait. That variant is fixed and the procedure is repeated for the remaining $k-1$ positions. (*Bottom*) The library of variants is created by performing saturation mutagenesis at *k* positions. The top variants are identified through screening. Those variants are randomly recombined to generate a second library, which is then screened to find the top design

Directed evolution (DE) is an iterative technique for designing molecules. It has been used to create proteins with increased stability [11], improved binding affinity [12], to design new protein folds [13], to change an enzyme’s substrate specificity [14] or ability to selectively synthesize enantiomeric products [4], and to study fitness landscapes [15], among others. Given an initial sequence, the primary steps in directed evolution are: (i) *random mutagenesis*, to create a library of variants; (ii) *screening*, to identify variants with the desired traits; and (iii) *amplification* of the best variants, to seed the next round. Each step can be performed in a variety of ways, giving rise to multiple options for performing DE. For example, the mutagenesis step can be performed one residue at a time, called a single mutation walk (Fig. 1-top), or simultaneously at multiple positions, followed by genetic recombination (Fig. 1-bottom). The key to the success of DE is that it performs what is in effect a parallel in vitro or in vivo search over designs that simultaneously *explores* the design space (via the mutagenesis step) while *exploiting* the information gained in previous rounds (via the amplification step). The exploratory aspect of DE is effectively a strategy for getting out of local optima on the underlying fitness landscape.

#### Machine learning-assisted directed protein evolution

**Fig. 2:**
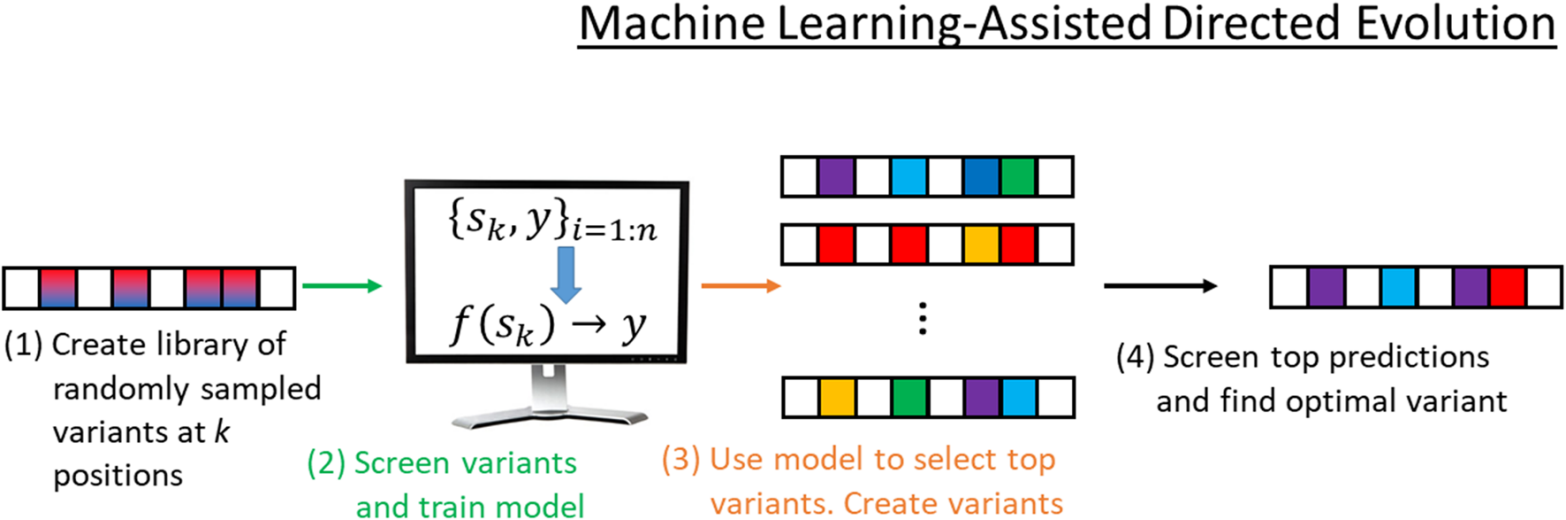
Machine learning-assisted directed evolution: The first step in ML-assisted DE is the same as for traditional DE (see Fig. 1). A library of variants is created via mutagenesis. Existing data, $\mathcal {S}=\{s_k,y\}_{i=1:n}$ are used to train a classifier or regression model, $f(s_k)\rightarrow y$, which is then used to rank variants via an in silico screen. The top variants are then synthesized/cloned and screened using in vitro or in vivo assays. The data from the *i*th round is added to $\mathcal {S}$ and used in subsequent DE rounds

While effective, the mutagenesis, screening, and amplification steps in DE are expensive and time-consuming, relative to in silico screens using statistical models or tools such as FoldX [10]. In an effort to reduce these experimental demands, a Machine learning-assisted approach to DE was introduced recently [4]. This ML-assisted form of DE is summarized in Fig. 2. The key difference between traditional and ML-assisted DE is that the data generated during screening are used to (re)train a model that is capable of predicting the property of interest for a given sequence. The model, $\hat{f}$, may be a classifier or regression model and acts as a surrogate for the true function, *f* (i.e., the one used by nature). The model can thus be used to perform an in silico screen over designs. Promising designs are then synthesized/cloned and screened in the lab. The key assumption made by ML-assisted DE is that the cost of performing an in silico screen using $\hat{f}$ is much lower than running wet-lab experiments (i.e., evaluating *f*). This assumption is almost always valid.

Like rational design, ML-assisted DE uses computational models, but the nature of those models is rather different. For one, the models used in ML-assisted DE make predictions corresponding to the quantity measured in the screening step, whereas the models used in rational design tend to be based on physical or statistical energy functions, and are therefore making predictions about the energetic favor of the design. Second, the models used in ML-assisted DE are updated after each DE round to incorporate the new screening data, and thus adapt to protein-specific experimental observations. The models used in rational design, in contrast, are typically fixed. Finally, the models used in ML-assisted DE are myopic, in the sense that they only consider the relationship between a small subset of sequence positions (ex. a binding site) and the measured quantity. The models used in rational design, in contrast, generally consider the *entire* sequence, and are thus better suited to filtering energetically unfavorable designs. The technique introduced in this paper seeks to combine the strengths of both methods; our method uses a fitness model that adapts to the experimental data, but also considers the favor of the mutations across the entire sequence.

### Bayesian optimization

Bayesian optimization [6] is an iterative technique for optimizing black-box (i.e., unknown) objective functions. It is frequently used when the objective function is expensive to evaluate, such as in the context of hyper-parameter optimization in deep learning (ex [16]). Protein design is a quintessential black-box optimization problem with an expensive objective function, and so it is a natural candidate for Bayesian optimization, including in the context of ML-assisted DE (ex. [17]).

In the Bayesian optimization framework, the objective function *f* is unknown, and so it is modeled as a random function with a suitably defined prior, *P*(*f*). At the beginning of each iteration, an algorithm known as the *acquisition function* [18] selects one or more candidate designs to evaluate, based on *P*(*f*). The resulting data are used to compute a posterior distribution over *f*, which becomes the prior for the next round. Typical choices for priors/posteriors include Gaussian processes [19] and Tree-structured Parzen estimators [16]. In the context of this paper, *f* is the function that maps protein sequences to an experimentally measured property (eg. fitness or binding affinity/activity), and our goal is to find $s^*={{\,\mathrm{arg\,max}\,}}_{s\in S}f(s)$, where *S* is the space of sequences. Naturally, the evaluation of *f* is expensive, because it requires the previously described DE mutagenesis and screening steps, but a surrogate function, $\hat{f}\sim P(f)$ can be used to perform in silico screens.

A variety of acquisition functions have been proposed, including: expected improvement (EI), upper (or lower) confidence bounds (UCB), Thompson sampling [20], and probability of improvement (PI). In general, an acquisition function defines some trade-off between *exploring* the design space, and simply selecting the point that has the best expected value under the posterior (aka *exploitation*). Our proposed approach uses custom acquisition functions that consider whether a given sequence resembles proteins observed in nature [21–23], in addition to the usual considerations of exploration and exploitation. Computationally, this is implemented using a regularization term, as defined in “Methods” section. We evaluated two types of regularization terms: (i) an evolutionary factor calculated using generative models of protein sequences, and (ii) a structure-based factor calculated using the program FoldX.

### Generative modeling of protein sequences

The statistical properties of protein sequences found in nature have been optimized through natural evolution to ensure that they have a full range of physical, chemical, and biological properties to function properly in a complex cellular environment. Therefore, one strategy for enforcing native-like properties in engineered proteins is to use a regularization term that penalizes designs that deviate significantly from the statistical patterns found in nature. To do this, we propose to use a generative model of protein sequences to calculate the regularization term. The primary model we investigate here is the Evolutionary Scale Model [7], a deep contextual transformer protein language model (TPLM). Within the field of Natural Language Processing, transformer models have become state-of-the-art over Recurrent Neural Networks (RNN). This is because the attention-based mechanism [24] used by Transformers allows the model to contextualize its focus on elements of a sequence it believes are most important. Transformer models not only capture long-range dependencies, but do so without the need to memorize the sequence from beginning to end. This increased efficiency lets this class of model handle larger training sets relative to RNNs. The transformer model used in this paper was trained on over 250 million protein sequences, and has been shown to learn representations for proteins that improve predictive performance over many tasks, including secondary structure and tertiary contact predictions.

In addition to the TPLM, we also evaluate two fold family-specific options—profile HMMs and Markov Random Fields (MRF), as generated by the gremlin algorithm [8] (see Additional file 1). HMM and MRF models can be learned from known sequences from a given fold family. The primary difference between these models is that HMMs make strong assumptions about the conditional independencies between residues. In particular, gremlin identifies and models both sequential and long-range dependencies. Either way, the models encode a joint distribution over residue types at each position in the primary sequence, *P*(*s*(1), ..., *s*(*n*)), which can be used to compute the probability (or related quantities, like log-odds) of given designs. We assume that any design with a high probability or log-odds under the generative model is native-like.

## Methods

### Data

#### Protein G B1 domain

Protein G is an antibody-binding protein expressed in groups C and G *Streptococcus* bacteria. The B1 domain of protein G (GB1) interacts with the Fc domain of immunoglobulins. We performed our experiments on data generated by Wu et al. [25], who performed saturation mutagenesis at four carefully chosen sites in GB1 in order to investigate the protein’s evolutionary landscape. The four chosen residues (V39, D40, G41, and V54) are collectively present in 12 of the protein’s top 20 pairwise epistatic interactions, meaning these sites are not just expected to contain evolutionarily favorable variants [26], but also those that are involved in interactions with each other.

The fitness criterion for their study was binding affinity to IgG-Fc. Experimental measurements were obtained for 149,361 out of 160,000 (i.e. $20^4$) possible variants at these four loci using mRNA display [27], followed by high-throughput Illumina sequencing. Briefly, this approach to measuring binding affinity works by first creating an input mRNA-protein fusion library from GB1 variants. This input library is then exposed to the GB1 binding target IgG-Fc. Any variant that binds to the target is subsequently sequenced for identification. By measuring the counts of each variant contained in the input library, $c^{\text {in}}$, and output “selected” library, $c^{\text {out}}$, the relative fitness *w* of the *i*th variant is calculated as follows:

1 $$\begin{aligned} w_i = \gamma \frac{c^{\text {in}}_i}{c^{\text {out}}_i} \end{aligned}$$

Here, $\gamma$ is a normalizing factor that ensures the wildtype sequence has fitness 1, and sequences with improved fitness are greater than 1. The range of fitness scores is from 0 to 8.76, with mean 0.08 (see Table 1). Only 3643 sequences ($\approx 2.4\%$) have fitness greater than 1.

**Table 1:** The mean, median, and variance for each scoring metric and each protein type

Protein	Metric	Mean	Median	Variance	(1)	(2)	(3)
GB1	(1) Fitness	0.08	0.003	0.16	1.0	− 0.09	− 0.25
	(2) TPLM Log-odds	11.55	11.49	3.80	− 0.09	1.0	− 0.01
	(3) FoldX $\Delta \Delta G$	9.42	8.39	27.93	− 0.25	− 0.01	1.0
BRCA1	(1) E3 ubiquitin ligase activity	0.63	0.60	0.17	1.0	0.31	− 0.38
	(2) TPLM log-odds	2.46	2.56	6.06	0.31	1.0	− 0.26
	(3) FoldX $\Delta \Delta G$	2.03	0.76	15.83	− 0.38	− 0.26	1.0
Spike	(1) ACE2 binding affinity	0.74	0.87	0.07	1.0	− 0.06	− 0.63
	(2) TPLM log-odds	2.62	2.73	0.42	− 0.06	1.0	0.11
	(3) FoldX $\Delta \Delta G$	2.51	1.48	14.36	− 0.63	0.11	1.0

The final three columns show spearman correlations between each respective score

#### BRCA1 RING domain

BRCA1 is a multi-domain protein that belongs to a family of tumor-suppressor genes. It contributes to this function through involvement in homology directed DNA repair, which undoes the genetic instability that underlies cancer development by fixing broken DNA strands. The RING domain of BRCA1 plays a critical role in this process by forming a heterodimer with fellow tumor suppressor BARD1 to constitute an E3 ubiquitin ligase, whose activity is responsible for this tumor suppressing function [28].

Starita et al. [29] investigated the functional effect of single site point mutations and deletions at BRCA1 residues 2-304 on E3 ubiquitin ligase activity. Using a phage display assay [30], they determined an E3 ubiquitin ligase activity score for 5154 total variant sequences. The activity was determined by calculating a relative change in abundance of each variant allele as a result of the assay. The scores were normalized so that the wildtype sequence had a score of 1, and nonfunctional sequences 0. After filtering the sequences based on quality, they found scores that ranged from nonfunctional to 2.8.

We used our approach on the Starita data to find BRCA1 RING domain sequences that maximize ubiquitin ligase activity, and thus provide insights into the factors governing sequence-activity relationships. Our experiments were limited to the mutations in residues 2-103 that passed the quality filter, which correspond to the RING domain coordinates. In total, this gave us 1388 variant sequences. Activity scores across these residues range from nonfunctional to 2.8, with an average score of 0.63 (see Table 1). Only 227 sequences ($\approx 15\%$) had activity scores greater than 1.

#### Spike protein receptor binding domain

The $\beta$-coronavirus SARS-CoV-2 is the virus responsible for the COVID-19 pandemic. The Spike glycoprotein is located on the virus’ surface, and plays a critical role in viral transmission. More specifically, the Spike receptor binding domain binds the human cell surface protein ACE2. Binding with ACE2 facilitates entry of the virus into the cell’s interior via membrane fusion or endocytosis.

Starr et al. [31] performed a deep mutational scan of the Spike glycoprotein using a yeast display platform to assess the effect of mutations in the receptor binding domain on ACE2 binding affinity. Their approach obtained measurements for each single site mutation within the protein binding domain [32]. In total, they tested 4020 variant sequences, and found binding affinities that ranged from − 4.84 to 0.3, where wildtype was normalized to have value 0 and higher scores are better. We removed knock-out variants, leaving all single-site point mutations, and re-scaled their affinities to fall within the unit interval. The re-scaled wiltype had score 0.94, and the average score was 0.74 (see Table 1). In total, 398 sequences ($\approx 11\%$) had an affinity greater than wildtype. We used our approach on the Starr data to find Spike receptor binding domain sequences that maximize affinity to ACE2, and thus provide insights into one of the factors governing host–virus interactions.

### Modeling

#### Evolutionary-based regularization factors

In order to obtain an evolutionary regularization term, we used pre-trained model ESM-1b made available through [7]. This transformer model is trained on all data available through the UniParc [33] database, and thus models *every* protein fold family represented therein. The model is trained using the following masked language model objective:

2 $$\begin{aligned} \mathcal {L}_{MLM} = \mathbb {E}_{s \sim S} \mathbb {E}_{M}\sum _{i \in M}-\log p(s(i) | s_{/M}) \end{aligned}$$

Each training sequence *s* is masked at a set of indices *M*. The model is trained by minimizing the negative log likelihood of each true amino acid *s*(*i*) given the masked sequence $s_{/M}$. In other words, the model must learn to use the surrounding sequence as context in order to predict the masked amino acids.

The model itself consists of 33 layers, and contains over 650 million parameters. While in principle the TPLM can be fine-tuned, we simply use it as-is. We use the model to obtain a log-odds score for a given protein design. To obtain this score, we provide as input a variant protein sequence, and the final layer of the TPLM outputs a logit for each possible amino acid at each position in the sequence. By summing over these logits for a given variant sequence, we obtain a TPLM-derived log-odds score. We calculated such log-odds scores for each variant in the available GB1, BRCA1, and Spike data.

In addition to using the TPLM, we also used MRF and HMM models to derive a fold-family specific regularization term. More detail on these models is given in Additional file 1.

#### Structure-based regularization factors

In order to obtain a structure-based regularization term, we used the FoldX protein modeling Suite. FoldX uses an empirical force field to calculate changes in energy associated with mutations to a protein’s amino acid sequence. It contains terms that account for Van der Waal’s interactions, solvation energies, energy due to Hydrogen bonding, energy due to water bridges, energy due to electrostatic interactions, and entropy due to dihedral angles and side chain conformations. For specific details of the FoldX force field, we refer the reader to [10].

For each protein tested, we obtained expected changes in Gibbs free energy (relative to the wildtype sequence) for each variant sequence. We followed the below protocol to obtain these energy calculations for each set of variant sequences:

1. Download a protein structure from the Protein Data Bank [34]. Table 2 shows the structures used for each protein tested.

**Table 2:** Protein structural data used for FoldX simulations

Protein	PDB ID	Experiment type	Resolution (Å)	Binding partner?
GB1	2GB1	Solution NMR	NA	No
BRCA1 RING Domain	1JM7	Solution NMR	NA	Yes
Spike PBR Domain	6M0J	X-ray diffraction	2.45	Yes

Binding partner refers to whether or not the structure includes the protein in complex with its binding partner
2. Repair the PDB structure using FoldX’s RepairPDB command. This fixes structures that have bad torsion angles, Van der Waal’s clashes, or total energy by rotating specific residues or side chains into more energetically favorable conformations.
3. Calculate the energy associated with introducing each mutation into the structure using FoldX’s BuildModel command. Calculations for each mutated sequence are done three times.

The final energy associated with each mutation is given by the average over the three FoldX runs. Mutations that are predicted to improve folding energy will have negative values, whereas positive values indicate an energetically less favorable mutation.

### Bayesian optimization for directed protein evolution

The Bayesian optimization is performed using Gaussian process (GP) regression as the prior over the unknown fitness or activity function, *f*. A GP requires a kernel, *K*, which describes the similarity between sequences $s_i$ and $s_j$. In our models, we use the squared exponential kernel (also known as the “Radial basis function”) given by:

3 $$\begin{aligned} K_{s_i,s_j} = \exp \bigg (-\frac{d(s_i,s_j)^2}{2\ell ^2}\bigg ) \end{aligned}$$

where $d(\cdot ,\cdot )$ is the Euclidean distance and $\ell$ the scalar length scale. We used a one-hot encoding of the variants. That is, each residue was assigned a length 20 vector, where each position corresponds to a specific amino acid. The position that corresponds to the residue present in the sequence takes value 1, and all others 0. Hyperparameter $\theta$ is optimized while fitting the GP to data by maximizing the log marginal likelihood:

4 $$\begin{aligned} \log p(y|\theta ) = -\frac{N}{2}\log 2\pi - \frac{1}{2} \log \text {det} |K+\sigma ^2I| - \frac{1}{2}y^T (K+\sigma ^2I)^{-1}y \end{aligned}$$

Since we use the squared exponential kernel in our experiments, length scale $\ell$ is the only hyperparameter. The term *y* is a vector of the given property (e.g. fitness) of *N* sequences, $\sigma ^2$ the variance of observations, and *I* is the $N \times N$ identity matrix. Once fitted, the GP encodes a distribution, $\mathcal {P}$, which is used to obtain a posterior mean function $\mu _{\mathcal {P}}(s_i)$ and variance over the unknown function *f*:

5 $$\begin{aligned} \mu _{\mathcal {P}}(s_i)&= \mathbb {E}\left[f(s_i)\right] = K_{s_i,s}(K+\sigma ^2I)^{-1}y \end{aligned}$$

6 $$\begin{aligned} \text {Var}[f(s_i)]&= K_{s_i,s_i} - K_{s_i,s}(K+\sigma ^2 I)^{-1}K_{s,s_i} \end{aligned}$$

$K_{s_i,s}$ refers to the row vector of kernel function values between sequence $s_i$ and all other sequences, denoted by subscript *s*. Additionally, $K_{s,s_i} = K_{s_i,s}^T$.

#### Regularized acquisition functions

The GP becomes the argument to an acquisition function, which is used to select sequences for wet-lab screening. The data produced via the screening step are used to update the GP for the next round. We performed experiments that used either the expected improvement (EI), probability of improvement (PI), or upper confidence bound (UCB) criteria as the acquisition function. Two versions of each acquisition function were considered: (i) the standard version, which is often used in Bayesian optimization, and (ii) a regularized form. The standard forms of EI, UCB, and PI are given by:

7 $$\begin{aligned} \text {EI}(s_i;\mathcal {P})&=\mathbb {E}_\mathcal {P}\big [\text {max}\big (0,f(s_i)-\mu _{\mathcal {P}}(s^+)\big )\big ] \end{aligned}$$

8 $$\begin{aligned} \text {PI}(s_i;\mathcal {P})&=P\big (f(s_i)>\mu _{\mathcal {P}}(s^+)\big ) \end{aligned}$$

9 $$\begin{aligned} \text {UCB}(s_i;\mathcal {P})&=\mu _{\mathcal {P}}(s_i)+\beta \sigma (s_i) \end{aligned}$$

where $s^+$ is the location of the (estimated) optimal posterior mean, $\beta$ a constant scaling factor (0.05 in our experiments), and $\sigma (\cdot )$ the standard deviation.

We also evaluated a *regularized* form of each acquisition function by scaling the standard version by a design-specific scaling factor, $\mathcal {F}(s;\mathcal {P})$. In our experiments, $\mathcal {F}$ refers to the evolution-based log-odds score obtained by either a TPLM, MRF, or profile HMM, or the structure-based $\Delta \Delta G$ calculated be FoldX, as described previously. Our regularized EI, PI, and UCB are defined as:

10 $$\begin{aligned} \text {EI}_{\mathcal {F}}(s_i;\mathcal {P})&= \text {EI}(s_i;\mathcal {P})\mathcal {F}(s_i) \end{aligned}$$

11 $$\begin{aligned} \text {PI}_{\mathcal {F}}(s_i;\mathcal {P})&= \text {PI}(s_i;\mathcal {P})\mathcal {F}(s_i) \end{aligned}$$

12 $$\begin{aligned} \text {UCB}_{\mathcal {F}}(s_i;\mathcal {P})&= \text {UCB}(s_i;\mathcal {P})\mathcal {F}(s_i) \end{aligned}$$

We will demonstrate in “Results” that this small modification to the acquisition function results in a substantial shift in the designs discovered via ML-assisted DE towards native-like designs, as expected.

### Directed evolution with machine learning and in silico traditional approaches

Our experiments contrast the performance of ‘standard’ ML-assisted DE (i.e., non-regularized) to the regularized version. We also compare the results to simulated forms of ‘traditional’ DE (i.e., without ML), as was also done in [4]. Since the BRCA1 and Spike protein data only include single-site mutations, we limit this analysis to the more exhaustive GB1 data. Specifically, we simulated both the single mutation walk and recombination versions of DE (see Fig. 1). We note that the single mutation walk approach is deterministic, given the starting sequence. With the single step, we start each trial with a randomly chosen sequence from the GB1 variant library. At each of positions 39, 40, 41, and 54, we observe the experimentally determined fitness values for all possible single-residue mutations. Having observed these mutations, we then fix in place the single-residue mutant which has the highest fitness. With this residue fixed, we then repeat this procedure for the remaining unfixed residue positions. Continuing in this manner, the trial ends when all residues have been fixed. All observed fitness values within a trial thus represent a DE determined fitness function approximation.

For the recombination method, we mimic saturation mutatgenesis experiments by starting with *n* randomly chosen sequences from the GB1 variant library. From these, we identify the top three sequences that have highest fitness (as was done in [4]), and use these sequences to perform recombination. A recombinant library is simulated in silico by computing the Cartesian product $S_{39} \times S_{40} \times S_{41} \times S_{54}$, where the set $S_m$ refers to the variant residues found at position *m* among the three highest fitness sequences in the initial random library. The resulting list of 4-tuples defines the recombinant library. Here, the DE fitness function approximation is given by observing fitness values for the *n* starting sequences as well as the recombined sequences.

## Results

In this section, we report the results of five approaches to performing DE: (i) single mutation walk (see Fig. 1-top); (ii) recombination (see Fig. 1-bottom); (iii) Bayesian optimization using standard acquisition functions (Eqs. 7–9), denoted by ‘GP + EI’, ‘GP + PI’, or ‘GP + UCB’; (iv) Bayesian optimization using evolution-based regularized acquisition functions with TPLM-derived log-odds, denoted by ‘GP + EI + TPLM’, ‘GP + PI + TPLM’, or ‘GP + UCB + TPLM’; and (v) Bayesian optimization using structure-based regularized acquisition functions with FoldX-derived $\Delta \Delta G$ values, denoted by ‘GP + EI + FoldX’, ‘GP + PI + FoldX’, or ‘GP + UCB + FoldX’. The regularized versions of each acquisition function used in (iv) and (v) is given by Eqs. 10–12. For simplicity, we focus our analysis on a subset of all experiments that we conducted. We include additional results briefly mentioned within the text in Additional file 1.

Each method was allowed to screen (e.g., obtain fitness values for) a total of 191 variants. This number was chosen to be similar to the number of sequences screened by the deterministic single mutant walk so that each method had similar experimental burden. Each model was initially trained on 20 randomly selected sequences. The small number of initial sequences simulates the scenario where the available fitness data is limited, prior to DE. The Bayesian optimization methods selected the top 19 sequences during each acquisition round. Each model is then updated with the experimentally measured fitness values for the chosen batch of 19 sequences, and this process is repeated for 9 batches (ie. 20 initial sequences plus 9 batches of 19 designs per batch, giving $20 + 19\times 9=191$ variants selected). We refer to a complete set of variant selection batches as a *trial*. We performed 100 total trials with each selection strategy with different random initial starting sequences. 20% of the data were held out for testing purposes (see Additional file 1).

### ML-assisted DE outperforms traditional DE

**Fig. 3:**
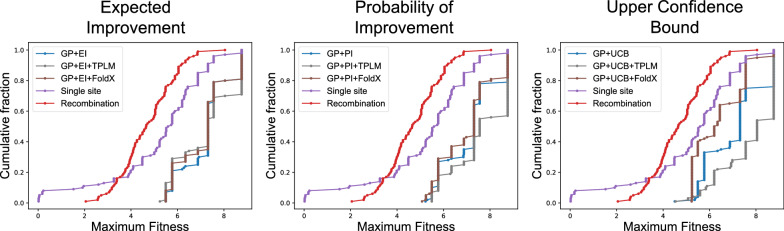
ML-assisted directed evolution techniques identify high fitness GB1 variants more frequently than simulated traditional DE approaches. Shown are the fraction of trials (y-axis) that reach less than or equal to a specified fitness (x-axis), where the selection criterion was either a simulated traditional DE approach, or standard or regularized EI, PI, and UCB was the acquisition function. (Left) Expected Improvement: The cumulative-weighted average fitness values are 7.25 for GP + EI + TPLM, 7.24 for GP + EI, and 7.16 for GP + EI + FoldX. (Middle) Probability of improvement: The cumulative-weighted average fitness values are 7.62 for GP + PI + TPLM, 7.17 for GP + PI, and 7.03 for GP + PI + FoldX. (Right) Upper confidence bound: The cumulative-weighted average fitness values are 7.76 for GP + UCB + TPLM, 7.10 for GP + UCB, and 6.38 for GP + UCB + FoldX. (All): The traditional single step and recombination approaches select variants with cumulative-weighted average fitness values of 5.22 and 4.71, respectively

Traditionally, DE techniques aim to identify sequences that score highly in one property. In Fig. 3 we demonstrate that ML-assisted DE outperforms simulated traditional approaches (i.e., single-mutant walk and recombination) when optimizing GB1 with respect to fitness. We observe this trend across all three forms of acquisition function tested (EI, PI, or UCB). On average, ML-assisted techniques (regularized and unregularized) identify a variant with fitness 7.22 (EI), 7.27 (PI), and 7.08 (UCB), whereas simulated traditional approaches identify a variant with fitness 4.97. The single mutant walk procedure finds a variant with maximum fitness 5.22, whereas recombination yields a variant with maximum fitness 4.71. Overall, we find that ML-assisted DE thus thus improves upon traditional approaches in designing high fitness GB1 variants by an average of 45%. This result is consistent with the findings in [4]. In Additional file 1: Fig. S1, we show that evolution-based regularization via gremlin and profile HMMs are also able to improve upon traditional DE techniques on the same GB1 variant selection task.

### Structure-based regularization usually leads to better designs

**Fig. 4:**
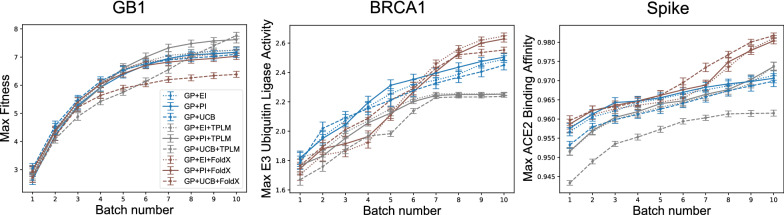
Regularization leads to better designs. Shown are the cumulative per batch scores for each protein averaged (± 1 SEM) over 100 trials. GP models were initialized with 20 randomly chosen sequences, and each batch consisted of 19 selected variants. Left: GP + UCB + TPLM selected the GB1 variant with highest average fitness (7.76), Middle: GP + EI + FoldX selected the BRCA1 variant with highest average E3 ubiquitin ligase activity (2.65), and Right: GP + UCB + FoldX selected the Spike variant with highest average ACE2 binding affinity (0.98)

Next, we investigated the effects of regularization and choice of acquisition function in the context of ML-assisted DE. Figure 4 shows the results of experiments on each protein using various regularized and unregularized acquisition functions. Overall, we find that structure-based regularization usually leads to better designs, and almost never hurts. The exceptions involve GB1. We note that the GB1 structure used in our experiments does not include the antibody, and so FoldX does not have a complete picture of the system being optimized.

Figure 4 also shows that the benefits of structure-based regularization vary according to the experimental budget. For example, if one were only able to perform four rounds, then the unregularized acquisition works better for BRCA1, but not for the other two proteins. Still, aside from the previously mentioned exception with GB1, structure-based regularization never hurts, given a sufficient number of rounds.

### Evolutionary-based regularization is unreliable

If a structure model is not available, it is natural to consider an evolutionary-based regularization term. That is, one based on a sequence model, like a transformer. However, we find that evolutionary-based regularization via the as-is ESM-1b TPLM is unreliable. As seen in Fig. 4, it does very well for GB1—outperforming both structure-based and the unregularized methods, but it underperforms for BRCA1 and Spike. This variability is perhaps expected, since a sequence model is obviously just an abstraction of a molecule. The TPLM apparently captures enough of the relevant information for the GB1 design task, but does not for BRCA1 or Spike. We also ran experiments that used gremlin and HMM regularized methods, but found that they did not perform much differently than unregularized methods (Additional file 1: Fig. S2-top). Together, these results suggest that one should obtain more consistent (in some cases, better) results using unregularized or structure-based ML-assisted DE compared to the evolutionary-based regularization methods we have tested.

### Correlation among traits

**Fig. 5:**
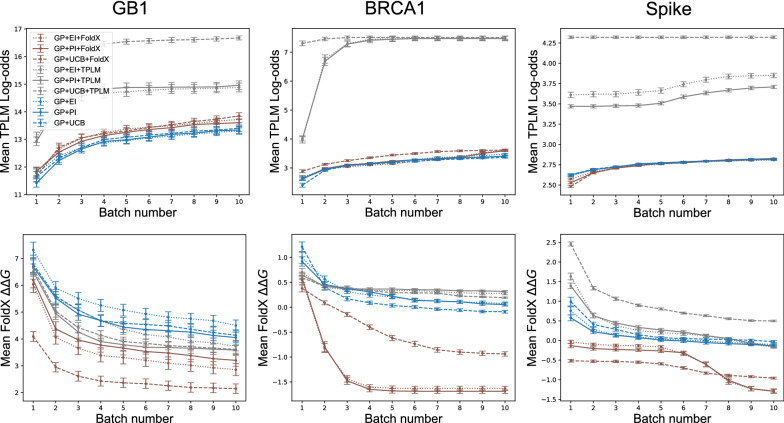
Evolution and structure-based regularization biases variant selections towards those that score favorably under multiple criteria. Shown are the regularization scores for variants selected for GB1 (Left), BRCA1 (Middle), and Spike (Right) under each selection criterion. As expected, variants selected by TPLM-regularized methods have higher log-odds under the TPLM than those selected from non-TPLM regularized methods (Top). Similarly, variants selected by FoldX regularized methods have lower $\Delta \Delta G$ values than those selected by non-FoldX methods (Bottom). The figures *also* show that TPLM-regularized methods tend to improve FoldX scores, and that FoldX-regularized methods tend to improve log-odds, indicating that there is some correlation between log-odds and thermodynamic stability

Naturally, the inclusion of a regularization term will bias variant selection towards designs that score favorably according to the regularization criteria, in addition to the objective value. This is seen clearly in Fig. 5, where the grey curves associated with the evolutionary bias achieve high log odds (top row), and the brown curves associated with the structural bias achieve low $\Delta \Delta G$ values (bottom row), as intended. The fact that the corresponding objective values are also high (see Fig. 4) simply indicates that the regularization terms generally do no harm. What is unexpected, however, is the fact that the blue and brown curves in the top row *also* rise, and the blue and grey curves in the bottom row *also* fall, even though those curves correspond to acquisition strategies that do not consider the quantity plotted on the *y* axis. This behavior reveals that the objective and regularization values carry some information about unmeasured traits. gremlin and HMM regularized acquisition functions reveal similar patterns (Additional file 1: Fig. S2-bottom)

### Evolution and structure-based regularization promotes site-specific exploration of unexplored sequence space

**Fig. 6:**
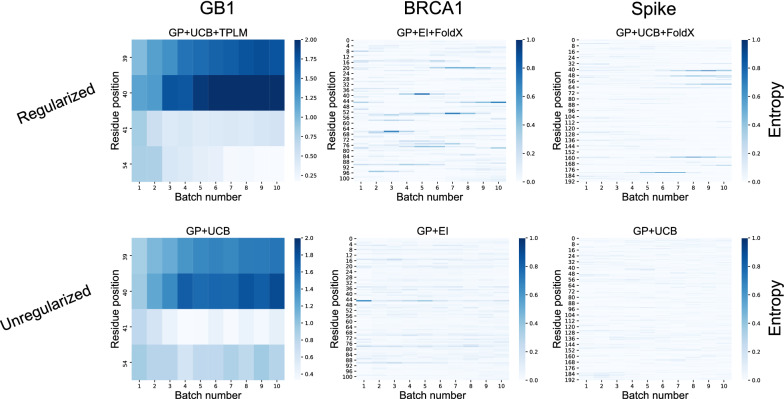
Bayesian selection techniques quickly identify informative sequence patterns. Shown are the per-batch average position-specific entropy of variant selections under the top scoring model for each protein. These include (Top) GP + UCB + TPLM for GB1, (Middle) GP + EI + FoldX for BRCA1, and (Bottom) GP + UCB + FoldX for Spike. Lighter squares denote low entropy decisions, meaning the model selects among fewer residue types at that position in that batch

Thus far, we have characterized variants selected by each ML-assisted DE technique in terms of their average fitness (GB1), measured activity (BRCA1), or binding affinity (Spike). We now consider the residue-specific behavior of choices made under each model. Figure 6 shows residue-specific entropy of variant selections with the best performing model for each protein. A high entropy block (dark blue) indicates that the method selects many different residue types at that position within a given batch. Low entropy blocks (light blue) indicate that the method selects only few residue types. The relative entropy of selections thus provides a sense of how the model explores sequence space, as well as the confidence the model has that a particular variant residue is informative.

Qualitatively, we notice that regularized methods for each protein have darker shading than their unregularized counterpart. This indicates that regularized methods explore more variant types at specific positions in each protein. With GB1 (Fig. 6-left), positions 39 and 40 have highest entropy for regularized and unregularized methods. The unregularized method has the least entropy at position 41, indicating that it has highest certainty at this position, whereas the regularized method has highest certainty at position 54. With both BRCA1 and Spike (Fig. 6-middle/right), there is generally much more light shading throughout the sequence, regardless of whether or not there is regularization. This is to be expected due simply to the larger number of positions that could be mutated using these protein data (recall that while the GB1 data includes all pairwise variants across four positions, these data include all single-site mutations across regions larger than 100 sequences each). Still, the regularized methods contain regions of darker shading, indicating a greater level of site-specific exploration. Given that the only difference between the methods shown for each protein is the presence or absence of regularization, it is the evolution or structure-based regularization that must drive this increased exploration at targeted positions.

**Fig. 7:**
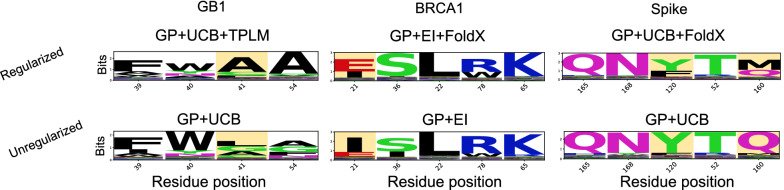
Evolutionary and structure-based regularization biases variant selection towards sequences with desirable properties. Shown are sequence logos for the best performing variant selection method along with their unregularized counterpart. All four residues are shown with the GB1 protein (Left), whereas the positions that correspond to variants with the top five true activity/binding affinity scores are shown for BRCA1 (Middle) and Spike (Right). Highlighted residues denote notable distinctions between the regularized and unregularized sequence selections

In Fig. 7, we show sequence logos obtained from the same models whose entropy are shown in Fig. 6. With GB1 (Fig. 7-left), the regularized method selected consensus sequence $\{\texttt {V}39\texttt {F},\texttt {D}40\texttt {W},\texttt {G}41\texttt {A},\texttt {V}54\texttt {A}\}$, whereas the unregularized method selected $\{\texttt {V}39\texttt {F},\texttt {D}40\texttt {W},\texttt {G}41\texttt {L},\texttt {V}54\texttt {A}\}$. While these sequences are similar, the single different amino acid selected at position 41 is meaningful in terms of the overall fitness of the design— $\{\texttt {V}39\texttt {F},\texttt {D}40\texttt {W},\texttt {G}41\texttt {A},\texttt {V}54\texttt {A}\}$ is the highest fitness variant in the data (8.76), whereas $\{\texttt {V}39\texttt {F},\texttt {D}40\texttt {W},\texttt {G}41\texttt {L},\texttt {V}54\texttt {A}\}$ ranks 443rd (3.66). With respect to BRCA1 (Fig. 7-middle) and Spike (Fig. 7-right), the sequence logos show amino acid selections for the positions where the top five scoring variants within each data set are located. For both of these proteins, the consensus sequences selected by the unregularized methods corresponds to the wildtype sequence. However, the best regularized method used for both proteins arrived at one consensus variant. With BRCA1, this corresponds to I21E, which is the highest scoring variant in the BRCA1 data. Additionally, the Spike variant Q120M selected by a FoldX regularized method has ACE2 binding affinity 0.18, which is the sixth highest scoring variant in the data set. Additionally, while Y is the consensus selection at position 120 for both models shown, we see that more trials that were regularized by FoldX selected variant Y120F, the third highest scoring variant, compared to the unregularized methods. Thus, regularized ML-assisted DE better identified the top scoring variant in the GB1 and BRCA1 data, and the third and sixth highest scoring variant in the Spike protein data compared to unregularized methods.

## Discussion

Our work extends ML-assisted DE via Bayesian optimization [17] by incorporating a regularization term into the acquisition function. The regularization term is intended to prevent the algorithm from optimizing the target property at the expense of unmeasured, but nevertheless important properties (ex. solubility, thermostability, etc). The results on the GB1 design task demonstrate that the inclusion of a regularization term can decrease the number of rounds needed to find high-fitness designs, relative to the unregularized version (Fig. 3), but the difference in performance does depend on which acquisition criterion is used (EI, PI, or UCB), and which regularization term is used (evolutionary or structure-based).

When our method is applied to more proteins, a clearer picture emerges. Given a sufficient number of rounds, structure-based regularization usually produces better designs, and only did harm in one configuration (GP + UCB + FoldX on GB1). In contrast, evolutionary-based regularization terms were seen to be unreliable; it only helped in two configurations (GP + PI + TPLM and GP + UCB + TPLM on GB1), but did poorly on BRCA1 and one configuration of Spike (GP + UCB + TPLM). Taken together, these results suggest that structure-based regularization using either EI or PI is beneficial or, at worst, neutral.

The one protein for which structure-based regularization using either EI or PI does not produce better designs than the unregularized version was GB1. Here, we note that GB1 was the one protein where the structure did not include the binding partner (see Table 2). That is, FoldX was not given relevant information, and so its predictions are less helpful as a guide during optimization. The structures used for the experiments on BRCA1 and Spike, in contrast, include the binding partner. It makes sense in this circumstance that FoldX will better determine the stability induced by a variant when in the presence of the binding partner—it simply has more relevant information.

As previously noted, evolution-based regularization by the TPLM is less reliable than its structure-based counterpart. One consideration that may contribute to this observation are the differences between the three data sets. For one, GB1 has the shortest sequence length (56) compared to BRCA1 (103) or Spike (193). It may be that the TPLM better captures interactions between sequence elements within smaller protein regions. The GB1 data is also more exhaustive than that for BRCA1 and Spike in that it contains all pairwise variants from four specific GB1 residues, whereas the others are limited to single-site mutations. More specifically, the four GB1 sites that were varied were chosen because they were predicted to be among the most involved in epistatic interactions [26]. That is, these residues are expected to contribute to long-range interactions between residues in GB1. The TPLM model we used was chosen because it is effective at encoding long-range dependencies within a protein sequence [7]. Thus, it may be that the GB1 data set is particularly well-suited to demonstrate the strengths of the TPLM. Another consideration is the TPLM itself. Recall that the TPLM uses unsupervised training across over 250 million different proteins to learn an effective representation for protein sequences. The model is thus very general, and not customized for a particular fold-family. Others have recently shown that a TPLM can be fine-tuned for a given fold family, and that a fine-tuned model can perform better compared to the general model on predictive tasks related to the tuned protein family [35]. An interesting direction for future work would be to fine-tune the TPLM model to determine whether doing so would improve the reliability of TPLM regularization in the context of ML-assisted DE task. If so, then our method may be more applicable to circumstances where structures are not available.

Finally, our results also demonstrate the benefits of a Bayesian approach to ML-assisted DE, as opposed to the approach introduced in [4]. When using the GB1 data (the only data set for which a head-to-head comparison is possible), we find that a Bayesian approach only required 191 fitness value acquisitions (20 initial observations plus 9 batches of 19) to identify designs with high fitness values. In contrast, the experiment in [4] required 470 initial observations plus a single batch of 100 to find similarly fit designs. This is a $67\%$ reduction in the number of sequences tested, which demonstrates the merits of Bayesian optimization in this context. The acquisition functions used in Bayesian optimization make a trade-off between exploration and exploitation of the domain, much like DE itself. Thus, ML-assisted DE via Bayesian Optimization effectively uses two forms of exploration and exploitation (one computational, one experimental). In contrast, the computational approach used in [4] is effectively pure exploitation. It is known that optimal regret bounds require a combination of exploration and exploitation [36], which may explain the advantage of Bayesian optimization in this context. As shown in Fig. 6, adding a regularization term to the acquisition function changes *where* the algorithm chooses to explore. This is seen by the differences in the entropy at select positions between the regularized and unregularized approaches. The regularized version tends to concentrate exploration at select residues, whereas unregularized methods select positions more uniformly. Notably, this trend is apparent in all regularized methods and all acquisition function types (Additional file 1: Figs. S3–S5). This difference in behavior leads to subtle changes in the final designs, as shown in Fig. 7, but regularization tends to produce the better design (Fig. 4).

## Conclusions

We have introduced a regularized approach to ML-assisted DE via Bayesian optimization. Two approaches were evaluated, one based on structure and the other based on evolutionary constraints. Our results suggest that structure-based regularization using an EI or PI acquisition function usually leads to better designs compared to unregularized approaches, and never hurts. In the absence of a structure model, it is natural to consider the use of a sequence-based regularization term. However, our results demonstrate that such terms do not lead to reliably better designs, at least for the specific proteins we considered. We plan to investigate fine-tuning the transformer model as part of future work, to see if doing so addresses this problem. Additionally, while we have shown how to introduce either evolutionary or structure-based regularization into a Bayesian DE framework, one can imagine combining the two into a single regularization term—we leave progress towards this to future work.

Previous research had demonstrated that ML-assisted DE can reduce the experimental burden, relative to traditional DE. Our results demonstrate that ML-assisted DE via Bayesian optimization decreases the experimental burden further, compared to the method in [4]. We also demonstrated that integrating a regularization term into the acquisition function can lead to better designs, and does so by concentrating exploration at select residues. We plan to investigate whether this insight might be helpful in fine-tuning the transformer model, analogous to the approach used to train Feedback Generative Adversarial Networks [37].

## Supplementary Information

**Additional file 1.** Additional methods and results including experiments regularized with gremlin and profile-HMM log-odds scores are located in Additional File 1.

## Data Availability

The GB1 variant fitness data is available through [25]. The GB1 fold family data is given by Pfam ID PF01378. The BRCA1 [29] and Spike [31] protein data are available through MaveDB [38], and are given respectively by IDs urn:mavedb:00000003-a-1 and urn:mavedb:00000044-a. Structure data is listed in Table 2, and can be download through the Protein Data Bank. The TPLM is described in [7] and the pre-trained ESM-1b model is available throught their GitHub at https://github.com/facebookresearch/esm. FoldX is available for free through an academic liscence at http://foldxsuite.crg.eu/.

## Abbreviations

DE: Directed evolution
ML: Machine learning
MRF: Markov Random Field
HMM: Hidden Markov Model
TPLM: Transformer Protein Language Model
GB1: Streptococcal protein G B1 domain
EI: Expected improvement
PI: Probability of improvement
UCB: Upper confidence bound
GP: Gaussian process
MSE: Mean squared error

## Standard Bayesian optimization approaches

GP + EI: Gaussian process with standard expected improvement acquisition
GP + PI: Gaussian process with standard probability of improvement acquisition
GP + UCB: Gaussian process with standard upper confidence bound acquisition

## TPLM-derived regularized Bayesian optimization approaches

GP + EI + TPLM: Gaussian process with TPLM log-odds regularized expected improvement acquisition
GP + PI + TPLM: Gaussian process with TPLM log-odds regularized probability of improvement acquisition
GP + UCB + TPLM: Gaussian process with TPLM log-odds regularized upper confidence bound acquisition

## FoldX-derived regularized Bayesian optimization approaches

GP + EI + FoldX: Gaussian process with FoldX calculated $\Delta \Delta G$ regularized expected improvement acquisition
GP + PI + FoldX: Gaussian process with FoldX calculated $\Delta \Delta G$ regularized probability of improvement acquisition
GP + UCB + FoldX: Gaussian process with FoldX calculated $\Delta \Delta G$ regularized upper confidence bound acquisition
