# Supplementary material for: Bayesian optimization with evolutionary and structure-based regularization for directed protein evolution
Source: Algorithms Mol Biol. 2021 Jul 1;16:13. doi: 10.1186/s13015-021-00195-4 (PMC8246133; doi:10.1186/s13015-021-00195-4)
Supplement: Supplementary file 1 — Additional file 1. Additional methods and results including experiments regularized with gremlin and profile-HMM log-odds scores are located in Additional File 1. [file 13015_2021_195_MOESM1_ESM.pdf]

RESEARCH

Bayesian Optimization with Evolutionary and Structure-Based Regularization for Directed Protein Evolution— Additional File 1

Trevor S. Frisby and Christopher James Langmead*

*Correspondence: cjl@cs.cmu.edu
Computational Biology Department, Carnegie Mellon University, 5000 Forbes Ave, 15213 Pittsburgh, PA, USA
Full list of author information is available at the end of the article

Additional methods

Sequences and notation

In our experiments, we use protein sequence data for proteins GB1, BRCA1, and Spike to identify variants that improve a targeted, experimentally measured quantity. In order to run our analyses, it is necessary to have a wildtype sequence for each of these proteins to use as a starting point. We use the following wildtype sequences for each of these proteins:

```
GB1:    MTYKLILNGKTLKGETTTEAVDAATAEKVFKQYANDNGVD
        GEWTYDDATKTFTVTE

BRCA1:  MDLSALRVEEVQNVINAMQKILECPICLELIKEPVSTKCD
        HIFCKFCMLKLLNQKKGPSQCPLCKNDITKRSLQESTRFS
        QLVEELLKIICAFQLDTGLEYAN

Spike:  NLCPFGEVFNATRFASVYAWNRKRISNCVADYSVLYNSAS
        FSTFKCYGVSPTKLNDLCFTNVYADSFVIRGDEVRQIAPG
        QTGKIADYNYKLPDDFTGCVIAWNSNNLDSKVGGNYNYLY
        RLFRKSNLKPFERDISTEIYQAGSTPCNGVEGFNCYFPLQ
        SYGFQPTNGVGYQPYRVVVLSFELLHAPATVCG
```

When referring to a sequence, the coordinates we use are with respect to the wildtype sequences above. That is, the first amino acid of each sequence corresponds to position 1, and so on. When we refer to a variant sequence, we use the notation 'X#Y,' where X is the wildtype amino acid, # is an integer denoting the position, and Y is the variant amino acid.

Fold family specific generative models of protein sequences

Our method can incorporate generative models of protein sequences for the fold family to which the target protein belongs. We evaluated two options for such models, (i) an MRF model learned using the GREMLIN algorithm [1], and (ii) a profile HMM, We downloaded the profile HMM [2] for the fold family to which GB1 belongs (Pfam id: PF01378) from the Pfam database [3]. We also downloaded the multiple sequence alignment that was used to train the HMM from Pfam, and then used the alignment to train the GREMLIN model. Thus, the GREMLIN and HMM models were trained from the same sequence data. We used these models to compute the log-odds of each design. These log-odds are used as a regularization factor in the Bayesian optimization The two models make different assumptions about the conditional independencies among the residues in the distribution over

GB1 sequences, and thus will output different log-odds scores for the same design, in general. Importantly, these are well-suited to training from limited sets of data, especially when compared to deep models. GREMLIN in particular learns a sparse model precisely to resist overfitting, and is thus better suited to learning from relatively small amounts of data.

Additional results

Evolutionary-based regularization of GB1 with GREMLIN and profile-HMM log-odds

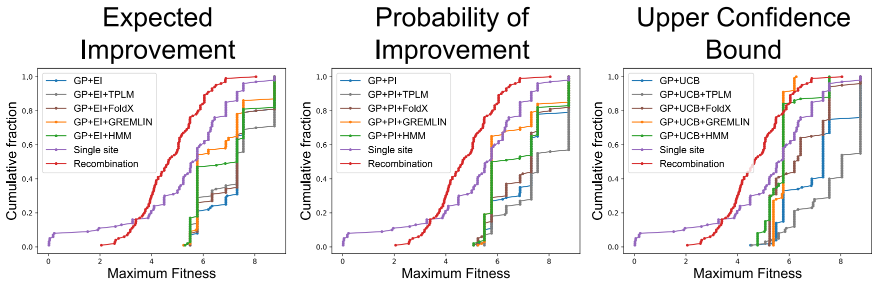

Figure S1 ML-assisted Directed Evolution techniques identify high fitness GB1 variants more frequently than simulated traditional DE approaches. Shown are the fraction of trials (y-axis) that reach less than or equal to a specified fitness (x-axis), where the selection criterion was either a simulated traditional DE approach, or standard or regularized EI **(Left)**, PI **(Middle)**, and UCB **(Right)** was the acquisition function. Experiments with methods regularized by gremlin and profile-HMM log-odds scores are shown alongside results depicted in Figure 3 in the main text.

Our experiments using GREMLIN and HMM regularized approaches follow the same sequential strategy used by experiments outlined in the main text. In Figure S1, we show how they compare to simulated traditional approaches, as well as those regularized by TPLM and FoldX. When EI or PI is used as the acquisition function, GREMLIN and HMM-based regularization typically identify variants with higher fitness relative to traditional approaches. Compared to the other ML-assisted methods, these approaches tend to identify variants with slightly lower fitness. When UCB is the acquisition function, we find that GREMLIN and HMM-based regularization outperform traditional approaches in roughly half of trials, but is outperformed by other ML-assisted approaches in most trials.

In Figure S2-top, we show the same results with GB1 from main text Figure 2 with the addition of GREMLIN and HMM regularized trials. While these additions do greatly improve upon wildtype GB1 fitness, with the exception of using structure-based regularization and UCB acquisition, other ML-assisted DE approaches, regularized or not, identify higher fitness GB1 variants. In Figure S2-bottom, we show that GREMLIN and HMM-based regularization has the intended effect of biasing variant selections towards those that have high log odds under each model. Putting together these results, when performing evolutionary-based regularization, TPLM is the best option for generative model compared to GREMLIN or profile-HMMs.

Additional sequence-space exploration experiments

In the main text, we describe how regularization induces site-specific exploration of unexplored sequence space. In the Discussion, we note that this behavior occurs for all regularization types, acquisition functions, and protein types that we

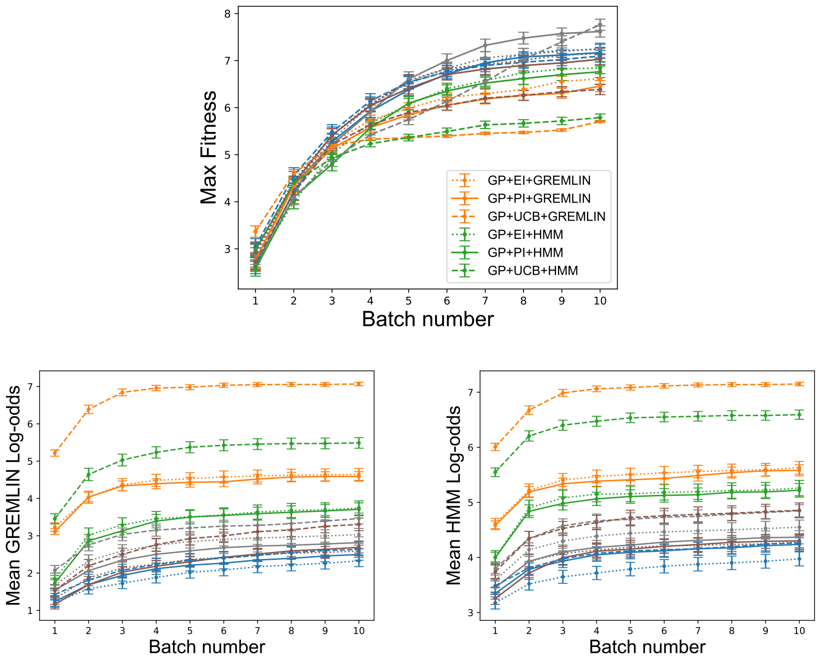

Figure S2 (Top) Regularization leads to better designs. Shown are the cumulative per batch scores for GB1 averaged ($\pm$ 1 SEM) over 100 trials. GP models were initialized with 20 randomly chosen sequences, and each batch consisted of 19 selected variants. Experiments with methods regularized by gremlin and profile-HMM log-odds scores are shown alongside results depicted in Figure 2-left in the main text. **(Bottom) Evolution and structure-based regularization biases variant selections towards those that score favorably under the regularization criterion.** Shown are the GREMLIN and HMM log-odds scores for variants selected from the GB1 ML-assisted DE experiments. Variants selected by methods regularized by both of these terms have high log-odds.

investigated. Figures S3-S5 show these results for each of GB1, BRCA1, and Spike, respectively. Columns from left to right show TPLM-based regularization, structure-based regularization, and unregularized approaches, and rows from top to bottom show experiments with UCB, EI, then PI acquisition functions. As described previously, we observe localized shading with greater intensity in regularized approaches compared to the unregularized ones. Even with GB1 where there is clearly more exploration at residues 40 and 39 compared to 41 and 54 regardless of regularization, there tends to be darker shading in the regularized approaches.

Predictions on unseen data

Table S1 Predictions made on held out data for each protein type, averaged across all regularization types. MSE refers to the mean square error over all predictions. Fitness/Activity/Affinity refers to the average true value for the predicted top sequence obtained for each method.

	GB1		BRCA1		Spike	
Regularization	MSE	Fitness	MSE	Activity	MSE	Affinity
Unregularized	1.96	4.99	0.14	1.47	0.04	0.93
TPLM	2.22	5.05	0.15	1.46	0.02	0.93
FoldX	1.89	5.04	0.14	1.35	0.04	0.92

In the main text, we characterize the batched variant sequence selections made by ML-assisted DE techniques with and without evolution or structure-based regularization. These selections allowed us to iteratively update GP models using sequences

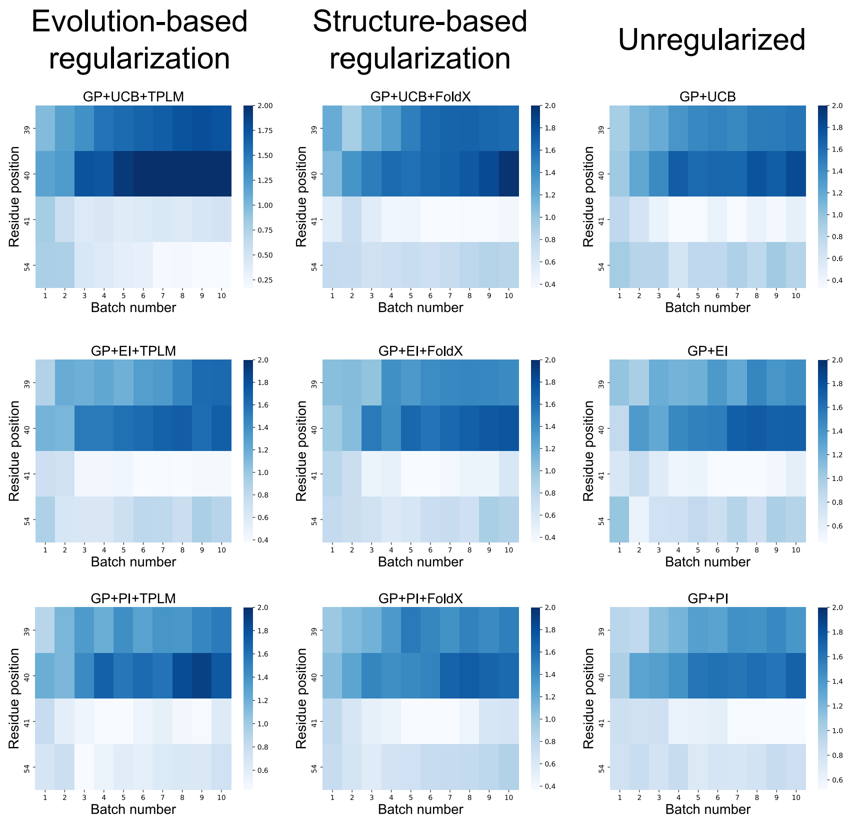

Figure S3 Bayesian selection techniques quickly identify informative sequence patterns. Shown are the per-batch average position-specific entropy of GB1 variant selections under each (un)regularized method.

that each model expected to be informative. To demonstrate the continued predictive capability of these models, we used them to predict the respective objectives of a held out test set for each protein. We emphasize that these sequences were never seen by the models during the iterative variant selection stage of each trial, and that they constitute a random subsample (20%) of the data for each protein.

For each protein, we used the models from the end of each trial to identify what they believe to be the best variant from the held out testing data. Table S1 shows the average mean squared error (MSE) averaged across all trials and acquisition types for each form of regularization. Additionally, it shows the average true value of this predicted best sequence. With GB1, we find that all models are high error, but do a good job at identifying a high fitness variant. With BRCA1, the models have better accuracy, and consistently identify a variant that improves upon wildtype E3 ubiquitin ligase activity. With Spike, all model types have good accuracy, and the top predicted sequence is generally comparable to wildtype ACE2 binding affinity. Thus, even when the models have relatively low accuracy, they are able to identify sequences that are comparable to or better than the wildtype sequence, similar to previous results [4].

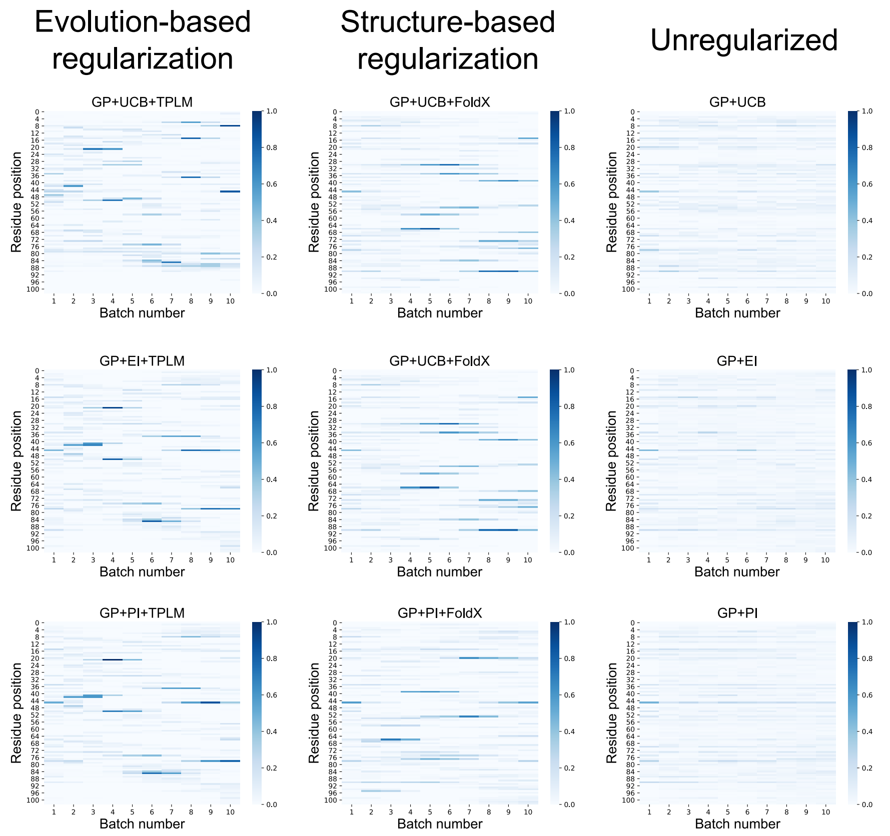

Figure S4 Bayesian selection techniques quickly identify informative sequence patterns. Shown are the per-batch average position-specific entropy of BRCA1 variant selections under each (un)regularized method.

Abbreviations not listed in main text

MRF-derived regularized Bayesian optimization approaches:

- **GP+EI+GREMLIN:** Gaussian process with GREMLIN log-odds regularized expected improvement acquisition
- **GP+PI+GREMLIN:** Gaussian process with GREMLIN log-odds regularized probability of improvement acquisition
- **GP+UCB+GREMLIN:** Gaussian process with GREMLIN log-odds regularized upper confidence bound acquisition

HMM-derived regularized Bayesian optimization approaches:

- **GP+EI+HMM:** Gaussian process with HMM log-odds regularized expected improvement acquisition
- **GP+PI+HMM:** Gaussian process with HMM log-odds regularized probability of improvement acquisition
- **GP+UCB+HMM:** Gaussian process with HMM log-odds regularized upper confidence bound acquisition

References

1. Balakrishnan, S., Kamisetty, H., Carbonell, J.C., Lee, S.I., C.J., L.: Learning Generative Models for Protein Fold Families. Proteins: Structure, Function, and Bioinformatics **79**(6), 1061–1078 (2011)
2. Krogh, A., Brown, M., Mian, I.S., Sjölander, K., Haussler, D.: Hidden Markov Models in Computational Biology. Journal of Molecular Biology **235**(5), 1501–1531 (1994). doi:10.1006/jmbi.1994.1104
3. El-Gebali, S., Mistry, J., Bateman, A., Eddy, S.R., Luciani, A., Potter, S.C., Qureshi, M., Richardson, L.J., Salazar, G.A., Smart, A., Sonnhammer, E.L.L., Hirsh, L., Paladin, L., Piovesan, D., Tosatto, S.C.E., Finn, R.D.: The Pfam protein families database in 2019. Nucleic Acids Research **47**(D1), 427–432 (2018). doi:10.1093/nar/gky995
4. Wu, Z., Kan, S.B.J., Lewis, R.D., Wittmann, B.J., Arnold, F.H.: Machine learning-assisted directed protein evolution with combinatorial libraries. Proceedings of the National Academy of Sciences **116**(18), 8852–8858

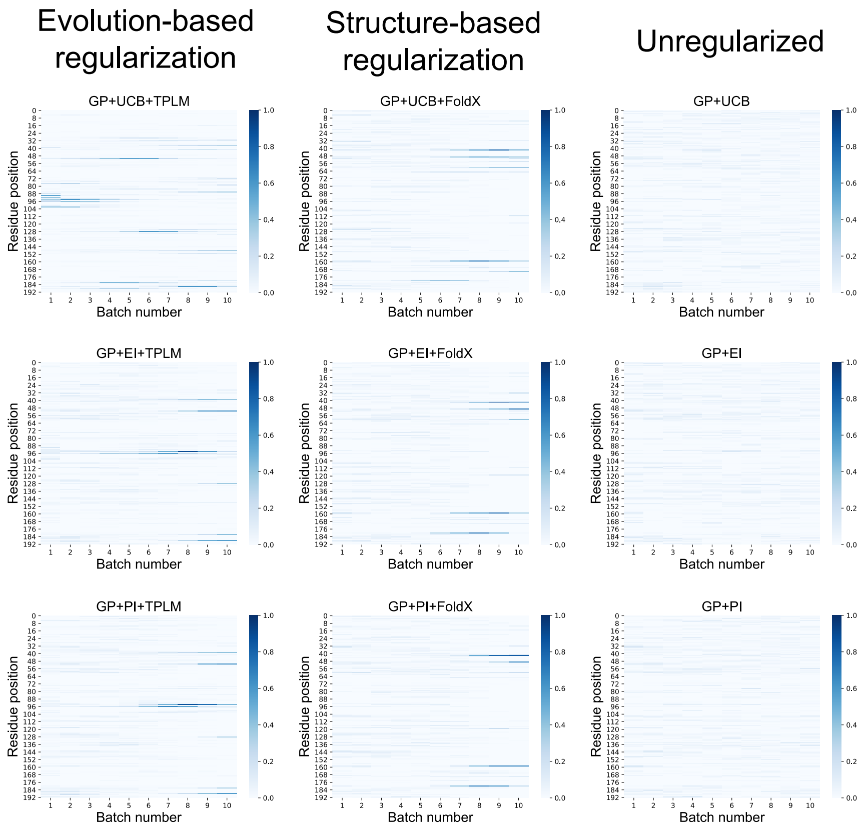

Figure S5 Bayesian selection techniques quickly identify informative sequence patterns. Shown are the per-batch average position-specific entropy of Spike variant selections under each (un)regularized method.

(2019)
